# Global challenges in oncologic imaging

**DOI:** 10.1186/1470-7330-14-S1-O1

**Published:** 2014-10-09

**Authors:** Hedvig Hricak

**Affiliations:** 1 Memorial Sloan Kettering Cancer Center, Department of Radiology, New York, USA

## 

Even as many cancers have been transformed from fatal to chronic diseases in developed countries, global cancer incidence and mortality have been rising [1]. While imaging is an essential tool in cancer care, the guidelines for its use are neither sufficient nor widely recognized. The needs for imaging equipment and training vary around the globe but are generally vast. Addressing these needs will require increased international cooperation. Conferences such as this, donation of equipment, “teach the teachers” programs, and ongoing education and consultation with experts via the Internet can all help. Most importantly, to have a long-lasting impact, cooperation must be tailored to local needs, engage local healthcare leaders, and build new generations of local leaders in oncologic imaging.

With few exceptions (most notably the United Kingdom), oncologic imaging has yet to mature as a subspecialty around the world. In the United States, only a handful of oncologic imaging fellowships exist, and radiologists in most hospitals are still organized around modalities or organ systems.

Just as pediatric radiology is not simply general radiology for small adults, oncologic imaging is not simply the identification of masses added to general body imaging. Sub-specialized expertise is required for optimal, clinically relevant interpretation. Oncologic imagers must understand all imaging modalities, even those they do not interpret primarily, to be full partners in multidisciplinary disease management teams. Yet oncologic imaging is not recognized as a separate section in board examinations, and most radiology residents do not perceive it as a potential career path. Formal acknowledgment of the value provided by dedicated oncologic imagers would help raise the visibility of this critical subspecialty.

Another challenge facing oncologic imaging is the need to standardize reports to ensure all key points are covered clearly for each specific kind of cancer [2]. As part of this effort, radiologists must learn to utilize quantitative rather than qualitative terms as much as possible. Not only should they use numerical scales to express diagnostic certainty, they must also learn to interpret an ever-growing array of quantitative imaging parameters [3].

Finally, while state-of-the-art equipment is widely available in the United States, the approval and dissemination of new molecular imaging probes has been frustratingly slow. To realize the true potential of oncologic imaging and assure its contribution to precision medicine, we need to expedite the validation, regulatory approval and dissemination of new tracers--particularly PET tracers, which have unparalleled molecular specificity. Radionuclide tracers are critical for characterizing tumor heterogeneity in vivo and are powerful predictive, prognostic and early response biomarkers. They can be used as companion diagnostics for targeted therapies and to assess pharmacodynamics/pharmacokinetics. Moreover, they can be integrated into theranostic agents that allow simultaneous diagnosis, treatment and treatment monitoring [4,5].

All these challenges may seem formidable, but as Alfred Einstein said, “in the middle of difficulty lies opportunity.” Despite its lack of recognition, oncologic imaging is poised to be part of a revolution in cancer care. With dedication, imagination and cooperation, there is no limit to what we can accomplish.
